# Disrupted Trajectories: COVID-19, Educational Track Mobility, and Socioeconomic Inequality in Dutch Secondary Education

**DOI:** 10.23889/ijpds.v11i4.3815

**Published:** 2026-07-06

**Authors:** Hekmat Alrouh

**Affiliations:** 1 Erasmus University Rotterdam, Netherlands

The COVID-19 pandemic disrupted secondary schooling across the Netherlands through prolonged school closures and hybrid instruction, raising concerns not only about learning loss but also about broader disruptions to students’ educational trajectories. Building on prior work on pandemic-related inequalities in final examination performance, this study examines two underexplored longitudinal outcomes: grade retention and track mobility. Using population-level administrative register data from Statistics Netherlands (CBS), covering all students in Dutch secondary education between 2013 and 2024 (N > 3,000,000), we reconstruct year-by-year educational trajectories. Each student-year is classified in terms of grade retention, upward mobility, downward mobility, and gap years. Together, these form the key construct of educational trajectory deviation, defined as any departure from the expected linear progression within a track.

The study distinguishes four periods: pre-pandemic (2013–2019), acute pandemic (2020), post-closure transition (2021–2022), and recovery (2023–2024). Generalised Estimating Equations with a logit link, clustered by student, model the probability of each outcome as a function of period, study year, and their interaction. We also estimate interactions with four dimensions of socioeconomic stratification: migration background, parental education, household income, and urbanicity.

We hypothesise that school closures increased grade retention and downward mobility, particularly among socioeconomically disadvantaged students, and that recovery has been unequal across groups. The findings contribute to stratification theory by showing how systemic disruptions propagate within tracked systems, and inform policy debates on equitable recovery from educational crises.

